# Multi-Scale Transcriptomics Redefining the Tumor Immune Microenvironment

**DOI:** 10.3390/biotech15010007

**Published:** 2026-01-15

**Authors:** Jing Sun, Yingxue Xiao, Lingling Xie, Dan Qin, Yue Zou, Yingying Liu, Yitong Zhai, Minyi Zhang, Tong Li, Youjin Hao, Bo Li

**Affiliations:** 1 Computational and Integrative Biology Group, College of Life Sciences, Chongqing Normal University, Chongqing 401331, China; 2023110513053@stu.cqnu.edu.cn (J.S.); 2023110513059@stu.cqnu.edu.cn (Y.X.); 2024110513055@stu.cqnu.edu.cn (L.X.); 2025110513077@stu.cqnu.edu.cn (Y.Z.); 2025110513054@stu.cqnu.edu.cn (Y.L.); 2025110513070@stu.cqnu.edu.cn (Y.Z.); 2025110513072@stu.cqnu.edu.cn (M.Z.); 2024210513012@stu.cqnu.edu.cn (T.L.); haoyoujin@hotmail.com (Y.H.); 2 Department of Biochemical and Cellular Pharmacology, Genentech Inc., One DNA Way, South San Francisco, CA 94080, USA; qin.dan@gene.com

**Keywords:** tumor immune microenvironment, bulk RNA sequencing, single-cell RNA sequencing, spatial transcriptomics, immune cell deconvolution, multi-dimensional integration

## Abstract

The tumor immune microenvironment (TIME) is closely involved in tumor initiation, malignant progression, immune escape, and response to immunotherapy. With the continued development of high-throughput sequencing technologies, transcriptomic approaches have become essential for examining the cellular and molecular features of the TIME. Bulk RNA sequencing offers tissue-level gene expression profiles and allows the estimation of immune cell composition through computational deconvolution. Single-cell RNA sequencing provides finer resolution, revealing cellular heterogeneity, lineage relationships, and functional states. Spatial transcriptomics (ST) retains the native anatomical context, making it possible to localize gene expression patterns and cell–cell interactions within intact tissues. These approaches, when considered together, have shifted TIME research from averaged measurements toward a more detailed and mechanistic understanding. This review summarizes the principles, applications and limitations of bulk, single-cell and spatial transcriptomic methods, highlighting emerging strategies for integrative analysis. Such multi-scale frameworks are increasingly important for studying immune dynamics and may contribute to the development of more precise biotechnological and immunotherapeutic strategies.

## 1. Introduction

The tumor immune microenvironment (TIME) is a highly dynamic and interactive ecosystem composed of tumor cells and immune infiltrates, together shaping tumor progression, immune evasion, and therapeutic responses [1]. As immunotherapies—such as immune checkpoint inhibitors [2,3]—continue to expand in clinical practice, it has become increasingly clear that understanding tumor immunity requires more than identifying broad immune signatures. Detailed knowledge of immune cell composition, functional states, and communication networks within the TIME is essential for defining treatment vulnerabilities. Accumulating evidence suggests that distinct myeloid subpopulations, including tumor-associated macrophages (TAMs), play an active role in shaping an immunosuppressive tumor immune microenvironment and driving T-cell dysfunction. These findings highlight the need for high-resolution characterization of immune heterogeneity to enable the identification of novel therapeutic targets [4]. Moreover, intratumoral myeloid cells exhibit pronounced plasticity, and their pathological polarization plays a critical role in shaping immunosuppressive states within the TIME, further highlighting the need for an in-depth understanding of immune cell functional states and reprogramming mechanisms [5].

A comprehensive depiction of TIME biology requires careful attention to its marked spatial and interindividual heterogeneity. Immune composition, activation states, and suppressive features differ not only between tumor types and patients but also across regions within the same tumor [6]. These variations reflect local tissue architecture and microenvironmental cues, making it difficult for conventional methods to capture the full complexity of immune organization. Traditional techniques such as flow cytometry (FACS) [7], mass cytometry (CyTOF), immunohistochemistry (IHC) [8], and multiplex immunofluorescence (mIF) serve as valuable tools for immune profiling, yet their ability to detect limited numbers of markers, combined with constraints in throughput and dimensionality, restricts their suitability for resolving complex immune landscapes [9]. Moreover, preserving spatial context while simultaneously measuring high-dimensional molecular information remains a key challenge for these approaches [10].

**Figure 1:**
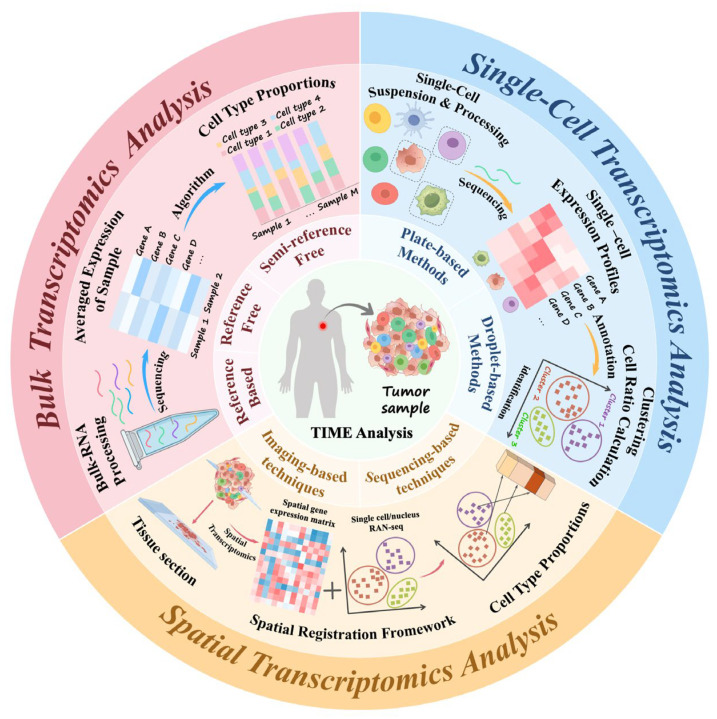
Three major transcriptomic strategies for characterizing the tumor immune microenvironment. Bulk RNA-seq, scRNA-seq and ST collectively illuminate the complexity and heterogeneity of the TIME at complementary levels of resolution. Bulk RNA-seq generates tissue-level, population-averaged gene expression profiles and enables inference of immune and stromal cell proportions using reference-based or semi-reference algorithms. The scRNA-seq dissects transcriptional variation at single-cell resolution, allowing cell-type identification through clustering and annotation, as well as quantification of cellular composition. ST preserves the native tissue architecture while capturing spatially resolved gene expression patterns, thereby revealing cellular localization and interaction networks, especially when integrated with complementary imaging or single-cell modalities.

The advent of high-throughput transcriptomic sequencing technologies has created new avenues for multidimensional dissection of the TIME [10], enabling unbiased, genome-wide characterization of cellular states and their underlying transcriptional regulatory networks [11]. Early bulk RNA sequencing (RNA-seq) approaches provided tissue-level summaries of immune-related transcriptional programs, laying a foundation for inferring immune infiltration patterns and defining molecular subtypes [12]. However, the mixed cellular signals inherent to bulk RNA-seq limit its ability to resolve single-cell heterogeneity. Advances in single-cell RNA sequencing (scRNA-seq) have since enabled high-resolution delineation of TIME composition and function, facilitating the discovery of previously unrecognized immune cell subpopulations and the elucidation of immune escape mechanisms [13,14]. More recently, spatial transcriptomics (ST) technologies have further expanded analytical capabilities by anchoring gene expression profiles within their tissue context, thereby providing spatial localization information and revealing histological patterns of cell–cell interactions [15]. Collectively, bulk RNA-seq, scRNA-seq and ST form a complementary methodological framework (Figure 1) that has advanced TIME research from population-averaged measurements to multi-scale, high-resolution, and spatially resolved analyses. Here, “multi-scale” refers to complementary biological resolutions rather than mathematical dimensionality or direct data-space compatibility.

Notably, the formation of the TIME is shaped not only by cellular composition but also by a wide range of finely regulated molecular processes, including the synthesis, modification, secretion, and degradation of signaling molecules [16]. Transcriptomic analyses primarily reflect gene expression programs that define cellular regulatory potential, whereas many rapid regulatory events at the protein level occur outside the transcriptional layer [17]. By governing genes involved in intracellular trafficking, degradation pathways, and signal transduction, transcriptional regulation remains tightly coupled to these molecular processes [18]. Consequently, transcriptomics provides a critical—though not exhaustive—perspective on TIME regulation, with particular strength in characterizing cellular states and functional programs.

Given the extensive use of transcriptomic approaches in tumor immunology, a systematic assessment of their underlying principles, applicability, and intrinsic limitations is warranted. This review summarizes the major technical features and research applications of bulk RNA-seq, scRNA-seq and ST in TIME studies. It also discusses emerging strategies for combining these data types to investigate immune regulation, understand determinants of immunotherapy response, and support precision oncology. By outlining a coherent methodological perspective, this review aims to assist future research in tumor immunology and contribute to its translation into clinical practice.

## 2. Bulk Transcriptomics and Immune Deconvolution

### 2.1. Overview of Technology and Methodology

As a core technological framework for profiling global gene expression patterns, bulk transcriptomics, including RNA-seq and DNA microarrays, has long played a foundational role in TIME research [19,20]. Among these approaches, RNA-seq is currently the most widely used bulk modality due to its high sensitivity, broad dynamic range, and capacity for largely unbiased, genome-wide quantification of polyadenylated transcripts [21]. Although microarrays depend on predefined probe sets, they retain value for analyses involving large, longitudinally accumulated cohorts and are still widely used in immunogenomics as well as in the development and benchmarking of cell deconvolution methods. Owing to the vast number of publicly available bulk transcriptomic datasets (e.g., The Cancer Genome Atlas (TCGA, Bethesda, MD, USA) and the International Cancer Genome Consortium (ICGC, Toronto, ON, Canada)), bulk profiling remains the most abundant and scalable source of expression data for investigations of the tumor immune microenvironment.

**Figure 2:**
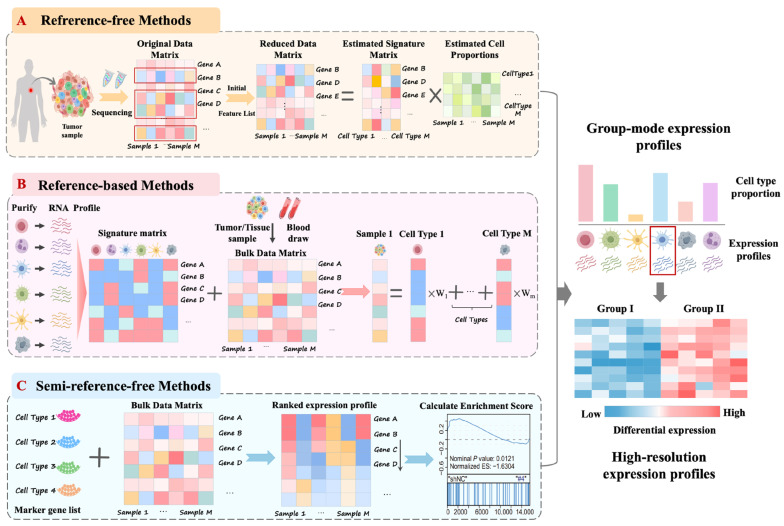
Major computational strategies for inferring cell composition from bulk transcriptomics data. Approaches to analyzing cell proportions using bulk transcriptomics data can be broadly categorized into two types: cell deconvolution based on expression matrix decomposition and cell abundance inference based on marker gene enrichment. (**A**) Reference-free methods perform unsupervised decomposition of bulk expression matrices to infer latent cell type specific expression signatures and relative proportions, based on general matrix or signal decomposition principles, without requiring external reference profiles; (**B**) Reference-based methods rely on feature gene matrices constructed from single-cell data to precisely estimate cellular composition within samples through regression modeling and similar approaches; (**C**) Cellular abundance inference based on marker gene enrichment primarily falls under Semi-reference-free methods. These combine limited prior information, such as marker gene list, with rank-sorting or enrichment scoring to estimate relative cellular abundances while maintaining flexibility.

From a systems-level perspective, bulk transcriptomic data support differential expression and functional enrichment analyses, allowing researchers to delineate immune-related molecular networks and regulatory programs that shape the TIME at a macroscopic scale [22,23]. Among its various applications, one of the most pivotal contributions of bulk transcriptomics to TIME research is its capacity to support cell composition inference, which remains a central objective in dissecting the immunological landscape of tumors (Method is shown in Figure 2, and some representative tools are listed in Table 1).

(1) Cell deconvolution strategies based on expression matrix decomposition. These approaches infer cell-type proportions or cell type-specific expression signatures by decomposing bulk tissue expression matrices into constituent cellular components [24,25,26]. This category can be broadly classified into two methodological subtypes:

① Reference-based methods: These techniques depend on predefined signature matrices derived from single-cell transcriptomic datasets or purified cell populations. By leveraging these external references, they enable precise estimation of cell-type abundances within heterogeneous tissues. Representative tools include CIBERSORT, MuSiC, and CPM [27,28,29,30].

② Reference-free methods: These approaches operate without external prior information. Rather than relying on predefined signatures, they infer latent cellular expression programs directly from bulk profiles using unsupervised techniques such as matrix factorization, principal component analysis, or independent component analysis. Notable examples include TOAST, SMC, Linseed, and deconf [31,32,33,34].

(2) Cell abundance inference strategies based on marker gene enrichment. These approaches, often also categorized as Semi-reference-free methods, do not rely on expression matrix decomposition. Instead, they estimate the relative abundance of immune cell types or their functional activity by computing enrichment scores derived from predefined marker gene sets. Representative tools in this category include MCP-counter and xCell, both of which infer cell-type abundance through robust gene set-based scoring frameworks [35,36]. Owing to their minimal model assumptions and strong resistance to technical noise, these methods are particularly well suited for research contexts in which immune cell type definitions are well established and marker genes are reliably characterized.

**Table 1:** Some representative tools used for Cell Composition Inferences.

Methods	Category	Platform	Algorithm	Version	Date	Ref.
CIBERSORT	RB	R (v4.3.1)	*v*-SVR	v0.1.0	2015	[27]
MuSiC	RB	R (v4.3.1)	*W*-CLS	v1.0.0	2019	[28]
CPM	RB	R (v4.3.1)	SVR	v0.1.6	2019	[29]
quanTIseq	RB	R (v4.3.1)	CLS	v1.10.0	2019	[30]
TOAST	RF	R (v4.3.1)	NMF/PCA	v1.20.0	2019	[31]
SMC	RF	MATLAB (R2020b)	Bayesian	v1.0.0	2017	[32]
Linseed	RF	R (v4.3.1)	Scoring	v0.99.3	2019	[33]
deconf	RF	R (v4.3.1)	NMF	v1.0.0	2010	[34]
MCP-counter	SMF	R (v4.3.1)	Scoring	v1.2.0	2016	[35]
Deblender	SMF	MATLAB (R2020b)	NMF	v1.0.0	2018	[37]
BisqueMarker	SMF	R (v4.3.1)	PCA	v1.0.5	2020	[38]
SCDC	RB	R (v4.3.1)	W-NNLS	v0.0.9	2021	[39]
ARIC	RB	Python (v3.8.20)	*W*-SVR	v1.0.1	2022	[40]
SQUID	RB	R (v4.3.1)	DWLS	v0.1.0	2023	[41]
GLDADec	SMF	Python (v3.8.0)	LDA	v1.0.0	2024	[42]

Notes: RB = Reference-based methods; RF = Reference-free methods; SMF = Semi-reference-free methods.

### 2.2. Application of Bulk Transcriptome-Based Cell Composition Inferences in TIME Research

Methods for inferring cellular composition from bulk transcriptomics have become indispensable for characterizing TIME features across large tumor cohorts [43]. By exploiting extensive bulk RNA-seq resources such as TCGA and ICGC, these approaches enable systematic quantification of infiltration patterns for a broad spectrum of immune cell populations—including CD8^+^ T cells, regulatory T cells (Tregs), natural killer (NK) cells, TAMs, and myeloid-derived suppressor cells (MDSCs). This, in turn, reveals cross-cancer variations in TIME composition and illuminates the immune ecological landscape at a macroscopic scale [24].

In pan-cancer analyses, the large-scale applications of CIBERSORT and other deconvolution algorithms to the TCGA datasets revealed a robust association between CD8^+^ T cell infiltration and favorable prognosis across multiple cancers [27]. Thorsson et al. integrated multiple cell inference tools to analyze over 10,000 tumor samples, proposing six pan-cancer immune subtypes (C1-C6) that demonstrate conserved immune phenotypic features across different cancer types within the TIME [44]. These studies underscore the critical value of bulk transcriptomics in constructing pan-cancer immune ecosystem maps.

In clinical prognostic studies, inferred cell composition is frequently integrated into Cox regression, Lasso, or various machine learning models to construct immune-related risk scores, which have been validated across multiple tumor types, including hepatocellular carcinoma [45] and breast cancer [46]. For example, analyses based on immune cell abundances estimated by algorithms such as TIMER [47] and EPIC [48] have shown that higher infiltration of B cells and CD8^+^ T cells is associated with significantly improved survival, whereas elevated macrophage infiltration predicts poor prognosis. Collectively, these findings highlight that immune infiltration profiles derived from bulk transcriptomics data possess robust clinical relevance and prognostic predictive value.

Furthermore, cell composition inference strategies have shown substantial promise in predicting responses to immunotherapy [49]. Using deconvolution-based analysis, Riaz et al. reported that melanoma patients treated with the PD-1 inhibitor nivolumab exhibited higher baseline CD8^+^ T-cell infiltration and demonstrated enhanced immune activation following treatment [50]. In the context of oncolytic virus therapy, bulk-based inference similarly captured hallmark features of immune remodeling—such as decreases in Treg abundance and increases in effector T-cell populations—providing valuable insights for tracking immune dynamics and refining therapeutic strategies [51].

Overall, bulk transcriptomics-based methods for inferring cellular composition have emerged as a critical bridge linking macroscopic gene expression patterns to the ecological characteristics of immune cells within the TIME. These approaches have demonstrated substantial value in immune subtype classification, prognostic stratification and the prediction of immunotherapy responses, because of their high scalability, low cost, and strong applicability to large clinical cohorts.

### 2.3. Advantages and Limitations of Cell Composition Inference Methods in TIME Research

Methods for inferring cellular composition from bulk transcriptomic data offer several notable advantages—including high efficiency, scalability, and strong clinical applicability—which position them as core analytical tools in TIME research [52]. Because these methods operate on RNA-seq or DNA microarray data, they are broadly compatible with diverse sample sources, such as fresh tissue, frozen tissue, and paraffin-embedded (FFPE) specimens. This versatility enables seamless integration with large clinical cohorts and supports key applications such as immune infiltration quantification, prognostic model construction, and immune subtype classification [53].

Despite their utility, deconvolution methods based on expression profile decomposition rely heavily on external reference matrices, which are typically derived from single-cell datasets or purified cell populations. As a result, they often fail to capture the full spectrum of heterogeneous and dynamically shifting immune cell states present within tumor tissues. For instance, TAMs frequently exhibit highly similar transcriptional signatures across M1- and M2-like states, making it difficult for algorithms to resolve functional subpopulations and thereby diminishing the accuracy of inferred cellular composition [54]. In contrast, marker gene enrichment strategies—although free from complex modeling assumptions—are strongly influenced by the choice of marker gene sets. When cell type boundaries are poorly defined or marker sets have substantial overlap, the resulting abundance estimates may lack specificity and show reduced discriminatory capacity.

Secondly, the inferred results are largely restricted to estimating the proportions of major immune cell categories, as bulk RNA-seq captures only the averaged expression across entire tissues. Such data cannot resolve finer functional states—for example, the degree of CD8^+^ T-cell exhaustion, the germinal center activity of B cells, or the activation status of NK cells. Moreover, strong expression signals from non-immune components—such as cancer cells, fibroblasts, and vascular endothelial cells—may dilute or even obscure immune-derived transcriptional patterns, thereby introducing additional bias into the estimated cell proportions. Although several algorithms (e.g., EPIC, quanTIseq) incorporate correction modules to account for stromal and tumor cell expression, fully mitigating this confounding influence remains a substantial challenge.

Overall, despite these limitations, bulk transcriptomics-based cell composition inference methods continue to serve as a critical link between conventional transcriptomic profiling and immunomapping analyses. Future methodological developments will likely focus on tighter integration with single-cell and spatial omics, which may help address current constraints in cellular resolution and spatial context. Such advances are expected to enable a more comprehensive and multilayered characterization of the TIME.

## 3. Single-Cell Transcriptomics for Dissecting Tumor Immune Microenvironment

### 3.1. Technical Background and Methodological Foundations

The TIME consists of diverse immune, stromal and tumor cell populations, whose intrinsic heterogeneity drives substantial variation in immune regulatory states and therapeutic responses. Traditional bulk transcriptomic approaches capture only tissue-averaged expression, making it difficult to resolve differences among cellular subpopulations, delineate functional state transitions, or characterize the roles of rare cell types. To achieve single-cell-level immune profiling and to dissect the architecture and dynamics of the TIME with greater precision, scRNA-seq has emerged and rapidly evolved into a core technology in TIME research [55,56].

**Figure 3:**
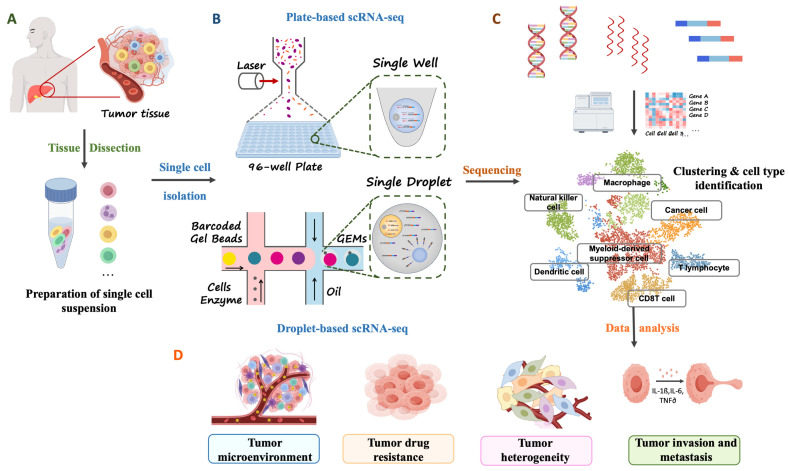
Experimental Workflow and Applications of scRNA-seq in Tumor Research. (**A**) Sample Preparation: Tumor tissues are dissociated into single-cell suspensions through enzymatic digestion and mechanical disaggregation, yielding individual cells suitable for downstream transcriptomic profiling. (**B**) Sequencing: Two major platforms are commonly used—plate-based methods, which provide high-coverage, full-length transcriptional information, and droplet-based methods, which enable high-throughput profiling of tens of thousands of cells per experiment. (**C**) Data Analysis: Raw sequencing data undergo quality control, normalization, dimensionality reduction, clustering, and cell-type annotation to generate high-resolution single-cell expression profiles of the tumor microenvironment (TME). (**D**) Applications: scRNA-seq is widely applied to delineate tumor microenvironmental composition, uncover drug resistance mechanisms, resolve cellular heterogeneity, and investigate tumor invasion and metastasis, thereby providing critical insights into cancer biology and therapeutic development.

The fundamental principle of scRNA-seq is to measure transcript abundance at single-cell resolution, enabling direct comparison of gene expression across individual cells. This capability allows researchers to identify rare immune subpopulations, delineate functional states—such as T-cell exhaustion and macrophage polarization—and reconstruct lineage relationships and differentiation trajectories. In doing so, scRNA-seq overcomes the inherent inability of bulk transcriptomic approaches to resolve cell-level heterogeneity [57]. Current scRNA-seq technologies largely fall into two major platforms—plate-based and droplet-based systems—which together define the dominant landscape of modern single-cell sequencing (Figure 3). Plate-based approaches (e.g., SMART-seq2) isolate single cells by FACS into multiwell plates and perform full-length transcript amplification. Their strengths—high transcript coverage, low technical noise, and precise gene-level quantification—make them particularly well suited for profiling rare cell populations or contexts requiring high-fidelity transcriptome characterization [58,59]. However, their relatively limited throughput constrains the number of cells analyzed per experiment. In contrast, droplet-based platforms (e.g., 10× Genomics) use microfluidic devices to coencapsulate individual cells with barcoded beads in oil droplets, enabling the profiling of tens of thousands to, in some cases, hundreds of thousands of cells in a single run [60,61]. This scalability has made droplet-based approaches widely adopted for systematically interrogating immune ecosystems, constructing comprehensive immune atlases, and identifying tumor-associated immune cell subsets at scale.

The data from scRNA-seq are characterized by high noise, substantial sparsity, and pronounced batch effects, necessitating a specialized analysis workflow to ensure accurate and interpretable results. Although different computational frameworks (e.g., Seurat (v5.4.0) [62], Scanpy (v1.9.8) [63], Harmony (v1.2.4) [64], fastMNN (v1.23.0) [65]) vary in implementation, the standard workflow generally includes the following components: (1) Quality control and cell filtering. Low-quality cells, dead cells, empty droplets, and doublets/multiplets are removed to retain biologically meaningful single-cell profiles. (2) Normalization and highly variable gene (HVG) selection. Normalization reduces technical variability, while HVG selection emphasizes biologically informative expression differences, ensuring that downstream analyses focus on meaningful signal rather than noise [66]. (3) Dimensionality reduction. Methods such as PCA or non-negative matrix factorization (NMF) extract major signal features, facilitating clustering and visualization. (4) Batch correction and data integration. Batch effects commonly arise in multi-patient, multi-experiment, or multi-platform datasets, requiring construction of a unified, comparable expression space. The sequence of correction steps depends on the integration strategy used. Approaches based on principal components or low-dimensional embeddings (e.g., Scanorama (v1.7.4) [67]) typically perform batch correction after dimensionality reduction. In contrast, methods operating on expression matrices or deep learning-based frameworks usually require pre-correction, or jointly conduct correction and dimensionality reduction within a single model. (5) Clustering and cell-type annotation. Clustering algorithms such as Louvain or Leiden identify cell populations with similar transcriptional profiles. Annotation then integrates marker gene expression, curated reference databases, or supervised classifiers to derive a comprehensive cellular composition and structural map of the TIME. To mitigate sparsity caused by dropout, imputation algorithms such as MAGIC (v3.0.0) [68], bayNorm (v1.5.14) [69], or scImpute (v0.0.8) [70] may be applied to stabilize downstream analyses when appropriate. Following annotation, researchers can further perform downstream analyses—including pseudotime trajectory inference [71], cell–cell communication analysis [72], or TCR/BCR repertoire reconstruction—to explore cellular ecological organization and immune regulatory mechanisms within the TIME.

### 3.2. Applications of Single-Cell Transcriptome Sequencing in TIME Research

Due to high resolution at the single-cell level, scRNA-seq has emerged as a key technology for deciphering the structure of the TIME and immune regulatory mechanisms. Its primary applications are concentrated in the following areas: (1) Analyzing immune cell composition and phenotypic heterogeneity. The scRNA-seq enables high-precision differentiation of immune subpopulations that are difficult to identify using bulk or traditional methods, demonstrating particular strength in T cells and myeloid cells. For instance, scRNA-seq revealed multiple stages of exhaustion and functional lineages within tumor-infiltrating CD8^+^ T cells, redefining the continuous state characteristics of T cell exhaustion [71,72]. Furthermore, TAMs do not follow a traditional M1/M2 dichotomy but instead exhibit a continuous spectrum of activation states encompassing pro-inflammatory, immunosuppressive, and tissue remodeling functional phenotypes [73].

(2) Reconstructing cellular differentiation trajectories and functional state transitions. Using pseudotime modeling and trajectory inference, scRNA-seq enables reconstruction of state-transition pathways for key immune cells within the TIME. For example, one study delineated a continuous trajectory from naïve T cells to effector and exhausted states, identifying a pre-exhaustion-like subpopulation associated with heightened sensitivity to immunotherapy [74]. In addition, scRNA-seq facilitates the detection and characterization of relatively rare but functionally critical immunosuppressive populations, including Tregs, MDSCs, and TAMs [75].

(3) Revealing cell–cell interactions and immune regulatory networks. Ligand–receptor interaction analyses based on scRNA-seq (e.g., CellPhoneDB (v5.0,1) [76], NicheNet (v2.2,0) [77]) enable the systematic interrogation of intercellular communication within the TIME. For example, studies have shown that CXCL chemokines secreted by tumor cells recruit Tregs into immunosuppressive niches, while TAMs promote immunosuppression and vascular remodeling through IL-10 and other immunoregulatory factors [78,79].

(4) Identifying key cell populations associated with immunotherapy response. scRNA-seq has been widely applied to dissect the cellular determinants of immune checkpoint inhibitor (ICI) efficacy. A growing body of evidence indicates that clinical responders frequently exhibit enrichment of Tcf7^+^ precursor-like CD8^+^ T cells, a population with self-renewal potential that can expand into effector cells upon treatment. The presence of this subpopulation is increasingly recognized as a critical prerequisite for achieving durable benefit from ICI therapy [80].

(5) Constructing multi-cancer immune atlases and enabling cross-cancer comparisons. With the accumulation of large-scale scRNA-seq datasets, researchers have established comprehensive immune atlases across multiple tumor types—including breast cancer, lung cancer, liver cancer, and melanoma [81,82]—thereby revealing inter-tumoral immune ecological differences and identifying shared immune regulatory modules across cancer types [83]. These atlases provide an essential foundation for defining tumor immune subtypes, characterizing cross-cancer immune landscapes, and developing pan-cancer immunological strategies.

### 3.3. Advantages and Limitations of Single-Cell Transcriptome Sequencing in TIME Research

Leveraging single-cell resolution and high-dimensional transcriptomic readouts, scRNA-seq provides unprecedented ability to resolve the cellular composition and functional states of the tumor immune microenvironment. It enables the identification of rare immune subpopulations, characterization of continuous state transitions, and reconstruction of immune cell differentiation trajectories. In doing so, scRNA-seq reveals layers of immune heterogeneity and regulatory features within the TIME that cannot be captured by traditional bulk approaches [84,85], thereby providing a robust molecular foundation for elucidating tumor immune-escape mechanisms and guiding immunotherapy optimization.

Despite its transformative analytical capabilities, scRNA-seq still faces several challenges in TIME studies. The resulting expression matrices are highly sparse and affected by substantial dropout events, which introduce uncertainty in quantifying lowly expressed genes and hinder accurate inference of cellular functional states [86]. Moreover, despite the steady decline in sequencing costs over recent years, overall experimental expenses, together with sensitivity to sample quality, tissue dissociation efficiency, and processing conditions, continue to limit the widespread application of scRNA-seq, particularly in large-scale clinical cohorts. More fundamentally, single-cell sequencing requires enzymatic and mechanical dissociation of tissues, which inevitably disrupts the native spatial architecture [87] and may lead to the loss or altered representation of certain cell types, such as stromal or fragile epithelial populations [84]. These issues collectively compromise the comprehensive reconstruction of the TIME [88].

To overcome these limitations and further extend its utility, future advancements should focus on multimodal integration and the reconstruction of spatial context. On one hand, combining scRNA-seq with ST can restore in situ cellular neighborhood relationships, thereby revealing the spatial organizational patterns of immune cells within the tumor microenvironment. On the other hand, increasingly sophisticated multimodal frameworks that integrate TCR/BCR sequencing, ATAC-seq and proteomic data are enabling researchers to dissect immune regulatory mechanisms across transcriptional, epigenetic, and clonal dimensions. In parallel, reducing batch effects and technical noise through algorithmic optimization, developing more robust models for cell-state characterization and implementing more cost-efficient sequencing platforms will collectively accelerate the adoption of scRNA-seq in both basic research and clinical practice. Taken together, despite current limitations, single-cell RNA sequencing enables high-resolution analysis of the tumor immune microenvironment and generates mechanistic insights that inform hypothesis-driven studies and immunotherapy design when supported by functional and clinical validation [89].

## 4. Spatial Transcriptomics and Immune Architecture in Tumor Research

### 4.1. Analytical Strategies and Technical Platforms

The core of spatial transcriptomics technology lies in its ability to perform detailed analysis of transcriptomic information while preserving the integrity of tissue spatial structure [88]. Unlike scRNA-seq, which requires dissociating tissue into single-cell suspensions, ST captures gene expression signals in situ within tissue, thereby revealing the spatial distribution patterns of cells within tissue coordinates and their molecular characteristics. This advantage endows it with unique scientific value and application potential for deciphering spatially dependent cell–cell interactions within the tissue-spatial-interaction framework.

**Figure 4:**
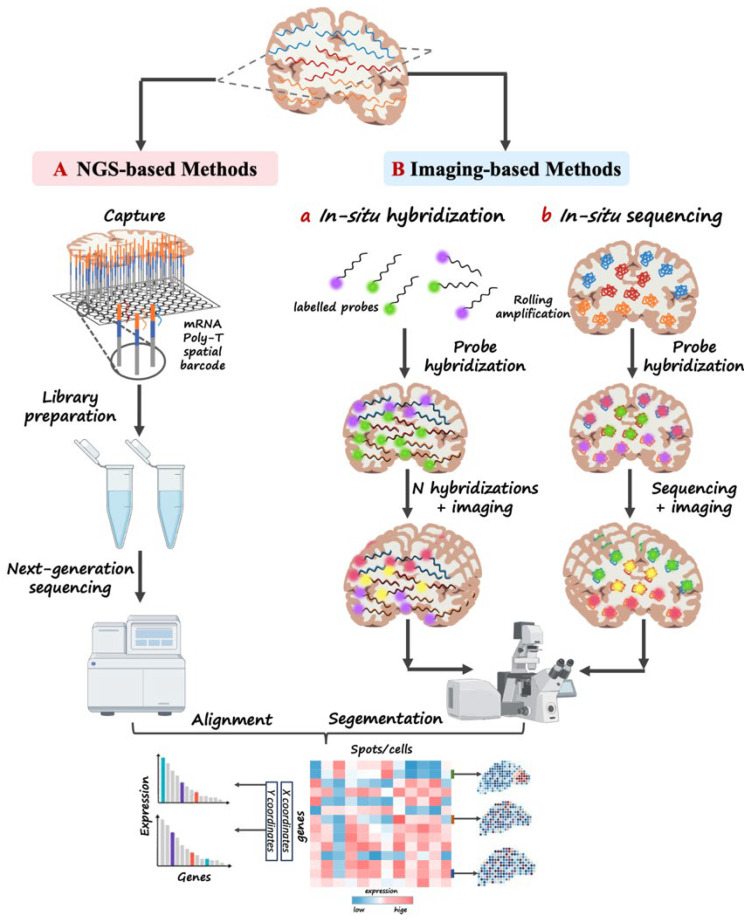
Two Major Technical Approaches in Spatial Transcriptomics: Principles and Workflows of NGS-Based and Imaging-Based Methods. (A) Sequencing-based methods: mRNA molecules within tissue sections are captured by spatially barcoded oligonucleotides printed on specialized chips, followed by reverse transcription, library preparation, and sequencing. Sequence reads are then computationally mapped back to their spatial coordinates to reconstruct spatial gene-expression landscapes. (B) Imaging-based methods: (a) In situ hybridization (ISH)-like approaches detect specific RNA species through iterative rounds of fluorescent probe hybridization and imaging, enabling high-resolution visualization of target transcripts within intact tissues. (b) In situ sequencing (ISS)-like approaches integrate probe hybridization, rolling-circle amplification, and imaging-based sequencing to simultaneously quantify multiple RNA molecules while preserving spatial context.

Current mainstream ST technologies can be broadly categorized into two types (Figure 4), NGS-based and imaging-based ST technologies [90].

NGS-based ST platforms that rely on capture probes—exemplified by 10× Genomics Visium—immobilize oligonucleotide probes carrying spatial barcodes onto glass slides, enabling in situ capture of mRNA molecules from tissue sections. After reverse transcription and sequencing, the resulting reads are computationally mapped back to their original spatial coordinates [91]. Owing to their mature workflows, broad transcriptome coverage, and compatibility with conventional RNA-seq analyses, these platforms represent the most widely adopted ST technologies to date. However, their spatial resolution remains constrained by spot size, limiting the ability to resolve gene expression patterns at single-cell or subcellular precision.

In contrast, imaging-based ST technologies, such as MERFISH [92], seqFISH+ [93], STARmap [94], and 10× Genomics Xenium [95], directly detect and localize RNA molecules within tissue sections through iterative rounds of fluorescent probe hybridization or in situ sequencing. Among these approaches, Xenium employs highly multiplexed, target-specific probe panels combined with cyclic fluorescence imaging to achieve in situ detection of predefined transcripts at subcellular resolution. This enables systematic interrogation of spatial interactions between tumor and immune cells while preserving native tissue architecture [96]. Owing to these features, Xenium has rapidly emerged as one of the standard tools for subcellular-resolution spatial mapping in cancer immunology. Overall, imaging-based spatial transcriptomics methods can achieve subcellular resolution and, in some cases, single-molecule resolution, thereby offering clear advantages for resolving fine-scale spatial interactions between tumor and immune cells. However, unlike sequencing-based spatial transcriptomics approaches, imaging-based technologies typically profile a predefined and limited set of transcripts, generally on the order of several hundred genes rather than the whole transcriptome, which constrains their capacity for unbiased transcriptome-wide discovery [97]. In addition, the requirements for advanced imaging instrumentation, optimized probe design, and complex signal-processing pipelines substantially increase the experimental and computational burden.

Recently, next-generation high-resolution ST platforms, such as Slide-seqV2 [98], HDST [99] and Stereo-seq [100], have rapidly gained prominence. By reducing probe spacing or leveraging DNA nanosphere arrays, these technologies obtain single-cell spatial resolution and retain coverage across large tissue areas. Their emergence has opened new avenues for generating high-resolution, tissue-wide spatial transcriptomic maps, substantially advancing the ability to chart the spatial architecture of tumors at unprecedented depth.

### 4.2. Applications of Spatiotemporal Transcriptomics in Tumor Microenvironment Research

ST addresses the limitations of bulk RNA-seq—which lacks spatial resolution—and scRNA-seq—which disrupts native tissue architecture—by preserving the in situ spatial coordinates of gene expression. This capability provides a novel framework for uncovering cellular distribution patterns, interaction networks, and functional states within the TIME [101].

In mapping immune spatial organization, ST can delineate the localization of distinct immune cell subsets across tumor regions, thereby revealing histological differences between immune-hot tumors and immune-cold tumors [102]. For instance, in pancreatic ductal adenocarcinoma (PDAC), Moncada et al. used ST to chart the spatial arrangement of immune and stromal cells and found that effector T cells are largely restricted to the tumor periphery, whereas fibroblasts form dense, barrier-like structures that hinder immune-cell penetration into the tumor core. This observation provides direct spatial evidence for the immune-exclusion phenotype characteristic of PDAC [103].

ST also plays a pivotal role in elucidating the structural niches within the TIME, enabling the identification of spatial subregions with distinct molecular features and functional states. For example, ST analysis of oral squamous cell carcinoma (OSCC) by Arora et al. revealed markedly divergent transcriptional programs between malignant cells located in the tumor core and those at the invasive front, each engaging in unique ligand–receptor interaction networks [104]. These findings indicate that tumor architecture is composed of organized spatial niches, each exhibiting distinct signaling patterns and biological functions, rather than being a random cellular aggregation.

At the level of clinical translation, ST is increasingly becoming a powerful tool for evaluating immunotherapy responses and discovering spatial biomarkers. Studies on non-small cell lung cancer (NSCLC) and melanoma have shown that responders to ICI therapy often exhibit spatial enrichment of immune-active populations—such as CD8^+^ effector T cells and NK cells—at the tumor periphery. In contrast, non-responders frequently demonstrate co-localization of immunosuppressive populations (e.g., Tregs and suppressive macrophages) with tumor cells, forming a local immune-rejecting microenvironment [105,106]. These spatial patterns provide critical insights into immune tolerance mechanisms and support the identification of spatially resolved biomarkers.

Overall, ST demonstrates substantial potential in mapping immune cell localization, revealing structured spatial niches, elucidating mechanisms of immune rejection, and predicting therapeutic responses, thereby establishing itself as an indispensable component of TIME research.

### 4.3. Advantages and Limitations of Spatiotemporal Transcriptomics

ST has introduced unprecedented spatial resolution to TIME research. By enabling transcriptomic measurements while preserving native tissue architecture, ST allows researchers to map the in situ spatial distribution and interaction patterns of immune, stromal, and tumor cells within tumor tissues [87]. With this capability, ST serves as a crucial bridge linking molecular features to tissue morphology, providing a powerful toolkit for dissecting immune infiltration patterns, identifying immune-exclusion barriers, and uncovering spatially relevant therapeutic targets.

Despite its transformative impact on understanding the spatial organization of the TIME, the application of ST remains constrained by several technical limitations. The most prominent challenge is limited spatial resolution: NGS-based platforms such as 10× Genomics Visium typically capture transcripts from tens of cells per spot, producing “local transcriptomes” that cannot resolve true single-cell heterogeneity [107]. Although next-generation platforms—including Slide-seqV2, HDST and Stereo-seq—have considerably enhanced resolution, these improvements come at the cost of increased detection noise, higher experimental complexity, and substantially greater expense, which collectively hinder their scalability for large-cohort studies [98].

In terms of expression detection, ST generally exhibits lower sensitivity than scRNA-seq. Insufficient mRNA capture efficiency and sequencing depth make it challenging to adequately capture low-abundance transcripts and rare immune cell subpopulations, such as specific T cell differentiation states or immunosuppressive myeloid cells [108]. Because fine-grained cellular states often drive immune dynamics, limitations in their detection directly constrain the reliability of predictive micromodels based solely on spatial transcriptomic data. Furthermore, differences in sample preparation, probe design and signal amplification strategies across platforms compromise result comparability, complicating cross-experiment sample integration. Computationally, ST data exhibits both high dimensionality and spatial dependency, requiring analysis workflows to identify spatial patterns while resolving mixed signals within spots. Common solutions to this problem involve combining algorithms such as dimensionality reduction, graph convolutional networks, or Bayesian modeling. However, the lack of a unified and robust standard analysis workflow remains a significant challenge in research [109]. This issue is particularly pronounced in highly heterogeneous tumor tissues: accurately distinguishing different cell types while maintaining spatial continuity is a core challenge for computational modeling. Importantly, ST primarily provides static, snapshot-based spatial expression maps. While spatial patterns may correlate with immune phenotypes, ST cannot directly capture key dynamic processes, such as immune receptor (TCR/BCR) clonal expansion, transient activation of signaling pathways, or therapy-induced immune reprogramming [110]. Consequently, ST data alone cannot fully reflect the temporal evolution of immune responses during tumor initiation, progression, or treatment.

Precisely for this reason, multi-dimensional integration has emerged as a necessary strategy for translating the spatial descriptive power of ST into a predictive modeling framework: Combining ST with scRNA-seq enables unified spatial localization and cell type annotation at single-cell resolution. Integrating it with bulk RNA-seq enhances sequencing depth and statistical robustness. The combined application of all three approaches (bulk + single-cell + spatial) holds promise for achieving panoramic analysis of TIME across breadth, depth, and spatial dimensions, thereby constructing a truly multi-level immune atlas. This multi-level integration strategy is emerging as a pivotal means for dissecting precision immunotherapy mechanisms and discovering targets, providing a more interpretable analytical framework for future spatial immunology.

## 5. Integrative Multi-Dimensional Transcriptome Framework

**Figure 5:**
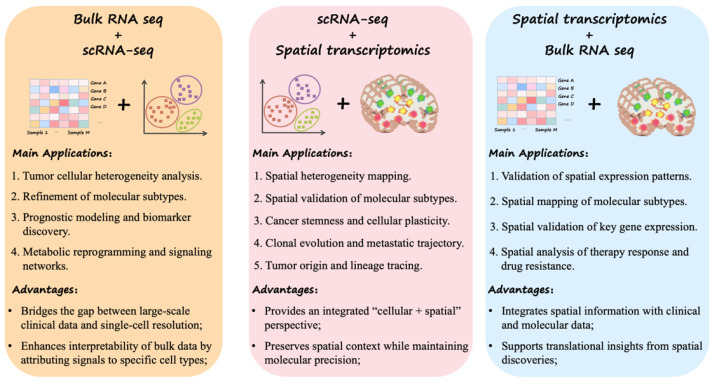
Applications of an integrated strategy combining bulk RNA seq, scRNA-seq and spatial transcriptomics in tumor research. Three representative combinations of transcriptomic technologies are widely used to interrogate molecular and spatial features of tumors across multiple scales: (i) Bulk RNA-seq + scRNA-seq: Using single-cell data as a reference to deconvolute bulk signals enables characterization of tumor and immune cell heterogeneity, refined molecular subtyping, prognostic model construction, and analyses of signaling pathway reprogramming. This approach effectively bridges large-scale clinical cohorts with single-cell-level resolution. (ii) scRNA-seq + ST: Integrating high-resolution molecular expression profiles with spatial context allows researchers to decipher spatial heterogeneity, validate spatially distinct molecular subtypes, and trace cellular lineages or metastatic trajectories within intact tissue architecture. (iii) ST + Bulk RNA-seq: Combining spatial expression maps with extensive bulk RNA-seq datasets facilitates validation of key spatial gene-expression patterns, supports analyses of treatment response and drug resistance, and strengthens the clinical translation of spatially derived biological insights.

With the continuous advancement of transcriptomic technologies, bulk RNA-seq, scRNA-seq and ST have become the three core approaches for TIME research, each illuminating a different layer of the tumor immune ecosystem—from the organismal to the single-cell and spatial levels (Table 2). Given the profound multi-level heterogeneity of the TIME in tissue architecture, cellular composition and signaling regulation, no single technology can fully capture its complexity or elucidate its biological underpinnings. In recent years, the combined use of multiple transcriptomic modalities has emerged as a major trend in tumor research. By building analytical bridges across population-scale, cellular-resolution, and spatially resolved datasets, multidimensional integration enables systematic exploration of the molecular mechanisms and spatial organizational principles underlying tumorigenesis and progression. This integrated framework provides a powerful new paradigm for multiscale interpretation of complex tumor tissues and for advancing comprehensive TIME characterization [103] (Figure 5). Representative computational tools and integration strategies are summarized in Table 3.

### 5.1. Reference-Based Integration of Bulk and Single-Cell Transcriptomics

The integration of bulk RNA-seq and scRNA-seq provides an effective strategy to bridge the gap between global gene-expression patterns and cellular heterogeneity. In practice, this integration is most commonly achieved through a reference-based projection framework, in which scRNA-seq data provide high-resolution, cell-type-resolved expression profiles for interpreting bulk transcriptomic measurements. Within this framework, cell-type-specific transcriptional signatures derived from scRNA-seq are mapped onto bulk RNA-seq data, enabling the inference of cellular composition and the reconstruction of cell-type-associated gene expression programs that are masked by bulk averaging [111].

Bulk transcriptomic profiling offers distinct advantages in terms of sequencing depth, technical robustness, and scalability to large patient cohorts, thereby capturing stable population-level gene expression landscapes with high statistical power. In contrast, scRNA-seq excels at resolving immune cell lineages, functional activation states, and fine-grained subpopulation structures, albeit typically in smaller cohorts and with increased technical noise. Reference-based deconvolution methods leverage the complementary strengths of these two modalities by anchoring bulk expression profiles to cell-type-resolved references, thereby restoring cellular interpretability to bulk RNA-seq data without sacrificing cohort size or clinical relevance.

A wide range of computational implementations have been developed under this conceptual framework, including CIBERSORT and its extended version CIBERSORTX [112], MuSiC [28], Bisque [38], and BayICE (v0.1.0) [113]. Although these methods are built on distinct statistical frameworks, including linear regression, weighted averaging, and Bayesian hierarchical modeling, they share a goal of leveraging scRNA-seq-derived reference profiles to estimate cell-type proportions and, where applicable, to infer cell-type-resolved expression from bulk transcriptomic data. The utility of this approach has been demonstrated across multiple cancer types. For instance, in colorectal cancer, immune cell signatures defined from scRNA-seq datasets have been projected onto large bulk RNA-seq cohorts using reference-based deconvolution strategies [26]. This integrative analysis has enabled robust estimation of immune infiltration levels and uncovered clinically relevant associations between specific T-cell functional states and patient prognosis. Such applications highlight the value of reference-based bulk–single-cell integration as a scalable and biologically interpretable strategy for dissecting immune heterogeneity in large clinical datasets [114,115,116].

### 5.2. Spatially Informed Decomposition Frameworks for Integrating scRNA-seq and Spatial Transcriptomics

The integration of single-cell RNA sequencing with spatial transcriptomics is predominantly realized through spatially informed signal decomposition frameworks, in which spatial coordinates are explicitly incorporated into the modeling process. Within this paradigm, cell-type-resolved expression profiles derived from scRNA-seq are leveraged as references to computationally decompose the mixed transcriptional signals captured within individual spatial transcriptomic spots, while preserving the native tissue architecture and spatial continuity.

By jointly modeling molecular abundance and spatial localization, this class of methods enables the reconstruction of spatial cell-type distributions and facilitates the inference of local co-occurrence and neighborhood relationships among immune, stromal, and malignant cell populations. Such spatially resolved deconvolution extends beyond simple cell-type mapping, allowing investigation of higher-order organizational principles that govern tissue microenvironments. Representative implementations of this framework include widely used spot deconvolution approaches such as RCTD (v1.2.0) [117], SPOTlight (v0.1.0) [118], cell2location (v0.1.4) [119]. These approaches employ diverse probabilistic designs and prior specifications to model cell-type abundance and transcriptional variability, yet all ultimately aim to anchor single-cell identities within spatial coordinates to disentangle the cellular composition of spatial transcriptomic data.

The biological value of this integrative strategy becomes particularly evident in complex immune niches. While scRNA-seq excels at delineating diverse immune cell subsets—such as functionally distinct B- and T-cell populations—and inferring potential ligand–receptor signaling programs, it inherently lacks spatial context. Conversely, spatial transcriptomics preserves tissue organization but cannot unambiguously assign transcripts to their cellular sources due to spot-level mixing. By combining these complementary modalities through spatially informed deconvolution, recent studies have demonstrated that specific ligand–receptor interactions are preferentially enriched within discrete microdomains, such as tertiary lymphoid structures. These analyses reveal spatially restricted immune activation programs that remain inaccessible to either technology in isolation [120]. Collectively, these analyses provide a refined framework for interrogating tumor–immune spatial interactions and for elucidating the organizational logic of the tumor microenvironment [109,110].

### 5.3. Cohort-Level Projection Frameworks for Integrating Bulk RNA-seq and Spatial Transcriptomics

The integration of bulk RNA sequencing with spatial transcriptomics primarily capitalizes on the complementarity between spatial resolution and global transcriptional depth and is most commonly implemented within a cohort-level projection and validation framework. Given the current technical constraints of spatial transcriptomic platforms—including limited detection sensitivity, reduced sequencing depth, and relatively small sample sizes—spatially resolved transcriptional patterns identified from individual tissue sections are often insufficient for robust population-level inference. To address this limitation, spatially defined expression programs are frequently projected onto large bulk RNA-seq cohorts, to evaluate their prevalence, inter-patient variability and clinical relevance.

In practice, this framework encompasses several closely related analytical strategies for linking spatial transcriptomic features to bulk RNA-seq data. One common approach projects gene-expression signatures derived from spatial transcriptomics, such as spatially variable genes, region-specific modules, or niche-associated programs, onto bulk transcriptomic data. This projection is typically achieved using enrichment- or regression-based methods, including GSVA (v2.0.7), single-sample GSEA, and linear modeling techniques [121]. In parallel, correlation-based frameworks offer a complementary strategy by directly modeling the concordance between bulk expression profiles and spatially resolved reference signatures. Some representative methods utilize predefined cell type or compartmental-specific expression profiles (e.g., SpatialDecon (v1.0.0)) to quantify their contributions to large amounts of RNA-seq data through frameworks based on correlation or constrained regression [122]. Rather than explicitly reconstructing spatial maps, these approaches provide a computationally efficient means of transferring spatially informed cellular or functional signals to population-scale transcriptomic datasets.

The biological relevance of this integrative paradigm has been demonstrated across multiple tumor contexts. For instance, spatial transcriptomic analyses have identified fibroblast-enriched tumor regions associated with immune exclusion, extracellular matrix remodeling, and immunosuppressive signaling. However, such findings are often derived from limited ST cohorts. By projecting or correlating fibroblast- or cancer-associated fibroblast-derived spatial signatures with bulk RNA-seq cohorts, the subsequent studies have shown that these immunosuppressive programs are widely conserved across patients and are strongly associated with unfavorable clinical outcomes. Collectively, cohort-level projection and correlation-based frameworks provide a critical bridge between spatial discovery and clinical generalization, enabling spatial transcriptomic insights to be contextualized within large-scale transcriptomic and outcome-linked datasets [123,124,125].

Overall, integrated analysis of bulk RNA-seq, scRNA-seq and ST offers a multi-level systems framework for tumor research, linking organism-level patterns with cellular and spatial organization. The combination of bulk and scRNA-seq focuses on complementing global signals with cellular heterogeneity, while the integration of scRNA-seq and ST emphasizes the coupling of cell types with spatial architecture through explicit spot deconvolution and spatial mapping of single-cell identities. Furthermore, the synergy between bulk and ST bridges the gap between large clinical cohorts and tissue spatial characteristics. In the future, as multi-omics integration algorithms continue to mature, spatial resolution steadily improves, and standardized data systems are established, these integration strategies will propel tumor research from two-dimensional transcriptomic analysis toward three-dimensional panoramic transcriptomic reconstruction. These efforts provide a more robust foundation for elucidating the spatial dynamics of tumorigenesis and development, as well as for developing precision treatment strategies [107].

## 6. Discussion

The emergence of multidimensional transcriptomic technologies has greatly expanded our ability to characterize tumor immune heterogeneity and immune regulatory mechanisms. From bulk RNA-seq to scRNA-seq and more recently ST, each modality contributes distinct advantages in resolution, coverage, and spatial context, collectively forming a multiscale analytical framework that spans organismal, cellular, and tissue dimensions. This review systematically summarizes the contributions and applications of these three technologies in TIME research, discusses their inherent limitations, and highlights the potential of multi-omics integration for constructing more comprehensive immune maps.

Despite the substantial progress enabled by these transcriptomic technologies, several challenges remain. Tumor tissues exhibit profound complexity in spatial organization, cellular composition, and transcriptional regulation, and single modality inevitably provides only a partial view of this landscape. Bulk RNA-seq offers high sequencing depth and stability but lacks cellular resolution; scRNA-seq captures cellular states and lineage relationships but disrupts tissue architecture; and ST preserves spatial context but continues to face limitations in resolution and sensitivity. Building a unified analytical framework capable of integrating data across these technological layers—and thus achieving coherent, cross-level interpretation—remains one of the central challenges in current TIME research.

Bulk RNA-seq, scRNA-seq and ST differ substantially in sample processing procedures, molecular capture mechanisms, noise characteristics, and data structures, making cross-experiment and cross-platform integration highly challenging. Achieving reliable integration therefore requires more robust computational methods and standardized analytical workflows. This issue is particularly pronounced in the ST domain, where technical heterogeneity—including variations in spatial resolution and differences in in situ detection chemistries—may introduce bias into analyses of spatial differential expression, cell localization, and spatial interaction networks. These issues underscore the urgent need for unified data-processing standards and evaluation frameworks.

From a methodological perspective, the apparent compatibility among bulk RNA-seq, scRNA-seq and ST is inherently conditional and task-dependent, rather than universal. Bulk RNA-seq is compatible with single-cell approaches primarily at the level of population-averaged expression patterns, cell-type composition inference, and cohort-scale statistical modeling, provided that appropriate assumptions regarding cellular composition and signal linearity are satisfied. In contrast, analyses that rely on intrinsic single-cell properties—such as fine-grained cell-state heterogeneity, lineage trajectories, or clonal dynamics—are fundamentally incompatible with bulk measurements due to irreversible signal averaging. Accordingly, bulk RNA-seq should be viewed not as a lower-resolution surrogate for single-cell profiling, but as a complementary modality optimized for robustness, scalability, and statistical power at the cohort level.

Moreover, the TIME is a highly dynamic system in which immune composition, cellular states, and spatial architecture undergo continuous remodeling in response to tumor progression, therapeutic pressure, and changes in the microenvironment. Current transcriptomic technologies predominantly provide “static snapshots,” making it difficult to directly assess dynamic processes such as immune cell migration, clonal expansion, or functional reprogramming. Future advances will require integrating time-series omics approaches with TCR/BCR sequencing, real-time imaging, and longitudinal sampling to capture the dynamic evolutionary trajectories of the tumor immune ecosystem with higher temporal resolution.

Looking forward, the increasing adoption of artificial intelligence, particularly large language models (LLMs) and foundation models, is expected to reshape computational paradigms for transcriptomic analysis and multimodal data integration. In contrast to traditional workflows that rely on hand-crafted features, predefined marker genes or task-specific models, foundation models emphasize representation learning, enabling the extraction of shared and transferable embeddings from large-scale transcriptomic datasets across modalities. In the context of TIME research, such models offer a promising framework for cross-modality alignment and scalability by learning unified latent representations across bulk RNA-seq, single-cell RNA sequencing, spatial transcriptomics and associated clinical metadata. This capability may facilitate more robust integration of population-scale and spatially resolved data, reduce reliance on manually curated references and improve generalizability across cohorts and tumor types. Meanwhile, applications of LLMs and foundation models in transcriptomics are still in their early stages, and key challenges related to interpretability, data bias, biological validation and reproducibility remain to be addressed.

As scRNA-seq and ST continue to mature, computational modeling advances and multi-omics integration frameworks become increasingly sophisticated, TIME research is poised to move beyond descriptive characterization toward the development of predictive and mechanistically driven systems models. Advances in high-resolution single-cell sequencing, subcellular-scale spatial omics, multimodal integration and large-scale immune mapping are collectively driving high-fidelity reconstruction of the tumor immune microenvironment. Ultimately, these advances will establish a strong theoretical foundation and provide extensive data resources for immunotherapy target discovery, robust efficacy prediction, and the design of precision immune interventions.

In summary, the integrated application of multidimensional transcriptomic technologies not only expands the depth and breadth of TIME research but also establishes a foundational framework for the future development of tumor immunology and precision medicine. A central challenge moving forward will be the construction of a unified, robust, and biologically interpretable multimodal analytical system capable of adapting to rapidly evolving technological landscapes—an endeavor that will shape the next stage of tumor immunomics research.

## 7. Conclusions

The integration of bulk RNA-seq, scRNA-seq and spatial transcriptomics provides a coherent framework for interrogating the tumor immune microenvironment across molecular, cellular and spatial scales. This review summarizes the principles, applications and limitations of three transcriptomic modalities in TIME research, with an emphasis on the scientific value and future potential of multi-omics integration. Advances in experimental platforms, algorithms, and AI-driven data analysis are shifting tumor immunology from two-dimensional expression profiling toward three-dimensional spatial ecosystems and dynamic evolutionary models. The long-term objective is to establish multidimensionally integrated, spatiotemporally resolved, and quantitatively interpretable tumor immune maps, thereby providing more precise molecular foundations for personalized immunotherapy, resistance mechanism studies, and the development of novel therapeutic strategies.

Continued progress in multi-omics technologies and computational methods will usher in a new era of spatially precise immunology, in which tumors will no longer be viewed as isolated lesions but as dynamic, modellable, and intervenable immune ecosystems. This transformation signifies not only a technological leap but also a fundamental paradigm shift in our understanding of tumor biology and immune regulation.

## Figures and Tables

**Table 2:** Advantages and limitations of three transcriptome sequencing techniques.

Technology Type	Key Features	Advantages	Limitations
Bulk RNA-seq	Obtain the overall gene expression levels of the tissue and screen for differentially expressed genes	(1) Technologically mature, cost-effective, and high-throughput; (2) Large sample size and robust data stability; (3) Can be closely linked to clinical characteristics and prognostic information; (4) Suitable for differential expression analysis and signaling pathway studies.	(1) Inability to distinguish expression contributions from different cell types; (2) Loss of cell heterogeneity information; (3) Difficulty in resolving minor or rare cell populations; (4) Significant susceptibility to variations in tissue sampling and cell proportions.
scRNA-seq	Analyzing Transcriptomic Features at the Single-Cell Level	(1) Reveals cellular heterogeneity and subpopulation structure; (2) Identifies rare cells and transient state cells; (3) Suitable for developmental trajectory and cell lineage tracing analysis; (4) Constructs intercellular communication networks.	(1) High cost and limited sequencing depth; (2) Sparse data, high noise levels, small sample size, and limited representativeness; (3) Difficulty in preserving spatial information and disruption of tissue structure.
Spatial transcriptomics	Transcriptome sequencing preserving spatial information of tissue sections.	(1) Reveals the spatial distribution characteristics of gene expression; (2) Preserves tissue morphology and structural information; (3) Identifies functional regions and intercellular spatial interactions; (4) Aids in understanding the spatial ecology of the tumor microenvironment.	(1) Limited resolution, with partial signal mixing; (2) Large data volume and complex analysis algorithms; (3) High cost and relatively low technological maturity; (4) Difficulty in direct matching with large clinical samples.

**Table 3:** Representative Tools for Integrative Transcriptomic Analysis of the TIME.

Integration	Tools	Core Strategy	Applications
Bulk RNA-seq + scRNA-seq	CIBERSORT MuSiC SCDC EPIC	Reference-based deconvolution using scRNA-seq-derived cell-type signatures	Immune composition profiling; Prognostic modeling; Cohort stratification.
scRNA-seq + ST	STdeconvolve Cell2location SPOTlight RCTD	Spot deconvolution by mapping single-cell profiles to spatial transcriptomic data	Resolution of spatial immune architecture; Investigation of tumor–immune spatial associations.
Bulk RNA-seq + ST	SpatialDecon Correlation-based frameworks	Validation of spatial gene-expression patterns using bulk RNA-seq cohorts	Validation of spatial biomarkers; Association of spatial features with clinical outcomes.

## Data Availability

The original contributions presented in this study are included in the article. Further inquiries can be directed to the corresponding author.
